# *Aspergillus flavus* and Aflatoxins (3rd Edition)

**DOI:** 10.3390/toxins17070326

**Published:** 2025-06-25

**Authors:** Tanvir Ahmad, Shihua Wang, Yang Liu

**Affiliations:** 1 School of Food Science and Engineering, Foshan University/National Technical Center (Foshan) for Quality Control of Famous and Special Agricultural Products (CAQS-GAP-KZZX043)/Guangdong Key Laboratory of Food Intelligent Manufacturing, Foshan 528231, China; tanvir@fosu.edu.cn; 2 Key Laboratory of Pathogenic Fungi and Mycotoxins of Fujian Province, College of Life Sciences, Fujian Agriculture and Forestry University, Fuzhou 350002, China

## 1. *Aspergillus flavus* and Aflatoxins

*Aspergillus flavus* is a saprophytic fungus commonly found in grain crops. It seriously threatens agriculture and food safety due to its ability to produce aflatoxins, potent secondary metabolites classified as Group I carcinogens by the International Agency for Research on Cancer [1]. *A. flavus* growth and aflatoxin contamination can occur both pre- and post-harvest, particularly under favorable environmental conditions, leading to substantial economic losses and health risks [2,3]. Beyond its toxigenic nature, *A. flavus* is also an opportunistic human pathogen capable of causing invasive aspergillosis and other serious infections [4].

Aflatoxins are among the most significant mycotoxins affecting food and feed safety. These naturally occurring toxic compounds are known for their hepatotoxic and immunosuppressive effects [5,6,7,8,9]. Aflatoxin contamination has emerged as a significant challenge in food safety and security in recent years [7,10]. While various mycotoxins (ochratoxin A, patulin, fumonisins, zearalenone, deoxynivalenol, and T-2 toxin) are of concern, aflatoxins remain the most prevalent and hazardous [11,12]. They frequently contaminate cereal grains and nuts at varying concentrations [13]. Prolonged exposure to aflatoxins can lead to acute and chronic health conditions in humans. Environmental, agronomic, and socioeconomic factors influence the risk of aflatoxin contamination.

Ensuring food safety requires thorough monitoring and effective processing to control mycotoxigenic fungi and mycotoxin levels in food products [14]. Various methods have been developed to detect and remove mycotoxins. However, their structural diversity, high stability, and low concentrations in food matrices demand robust, sensitive, and eco-friendly detection techniques [15]. Chromatographic methods such as HPLC, LC-MS/MS, and GC-MS/MS are widely employed, with LC-MS/MS favored for its multiplex detection capability [10]. Immunoassay-based tools like ELISA and biosensors also offer rapid and reliable screening [16,17]. In addition, novel detection techniques—such as electronic noses, proteomics, Raman spectroscopy, molecular diagnostics, and hyperspectral imaging—are emerging as promising alternatives [18,19,20,21].

Implementation of Hazard Analysis and Critical Control Points (HACCP)-based methods can help reduce aflatoxin contamination. While conventional methods can detoxify aflatoxins in food, increasing resistance in *A. flavus* and efficacy limitations highlight the need for new strategies that preserve food quality. Emerging approaches—such as polyphenols, enzymes, essential oils, plasma, and nanoparticles—offer enhanced detoxification potential, especially when combined. Microbial detoxification is also gaining attention as a safe and eco-friendly alternative [9,22,23,24]. Despite significant advances in detection methods, the mechanisms responsible for the effective *A. flavus* inhibition and aflatoxin detoxification remain poorly understood. This highlights the urgent need for integrated, multidisciplinary research approaches to address these challenges. Hence, this editorial summarizes the key findings from the Special Issue titled “*Aspergillus flavus* and Aflatoxins (3rd Edition)” (https://www.mdpi.com/journal/toxins/special_issues/4DCZ19EGC1, accessed on 4 April 2024), which includes 13 contributions (10 research articles and 3 reviews) from different groups of scientists around the world. The advances are discussed in five thematic areas: (a) aflatoxin detection technologies, (b) detoxification and post-harvest control strategies, (c) AFB_1_ toxicity and health effects, (d) fungal biology and aflatoxin biosynthesis regulation, and (e) host susceptibility and crop/public health risks. We believe this editorial will be a valuable resource for researchers working on mycotoxins and food safety.

## 2. Aflatoxins Detection Technologies

Recent innovations in aflatoxin detection technologies have paved the way for faster, more sensitive, and environmentally sustainable food safety solutions. Kourti et al. (contribution 12) introduced an immersible silicon photonic immunosensor for rapid, on-site detection of aflatoxin M_1_ (AFM_1_) in whole milk, offering a practical and high-performance tool for global food monitoring. The sensor employs dual U-shaped silicon nitride waveguides configured as Mach–Zehnder interferometers (MZIs), functionalized with AFM1-specific biomolecules. The system maintains high sensitivity by eliminating pumps and microfluidics while enhancing portability and ease of use. It achieves a detection limit of 20 pg/mL—surpassing European Commission thresholds for both infant (25 pg/mL) and adult (50 pg/mL) milk—and delivers accurate (86–112% recovery), reproducible (CV < 10%) results within 20 min, without the need for sample pretreatment.

Wu et al. (contribution 9) present an eco-friendly supramolecular platform for detecting aflatoxin B_1_ (AFB_1_), based on host–guest interactions using acyclic cucurbit [6] uril (acCB6). The study confirms a stable 1:1 complex between AFB_1_ and acCB6 in water, supported by ^1^H NMR, isothermal titration calorimetry, and molecular dynamics simulations. This host was integrated into a scaffold-assembled bioluminescent enzyme immunoassay (SA-BLEIA), enabling a solvent-free detection method with sensitivity comparable to standard assays. This approach addresses environmental concerns associated with conventional techniques and underscores the potential of supramolecular chemistry in advancing sustainable food safety diagnostics.

## 3. Detoxification and Post-Harvest Control Strategies

Cuccato et al. (contribution 8) evaluated the efficacy of two novel smectite-based mycotoxin detoxifying agents (SeOX and CHS) in mitigating the harmful effects of AFB_1_ in broiler chickens exposed to dietary AFB_1_ levels near EU regulatory limits (0.02 mg/kg). Both additives significantly reduced AFB_1_ bio-accessibility by ~30%, as evidenced by higher AFB_1_ excretion in treated groups compared to the AFB_1_-exposed control. This reduction correlated with diminished oxidative stress, including improved antioxidant capacity and reduced hepatic lipid peroxidation. While AFB_1_ alone upregulated CYP2A6 (a pro-oxidant enzyme) and suppressed Nrf2 (a regulator of antioxidant defenses), both MyDA treatments reversed these molecular changes, restoring redox balance. The additives showed no adverse effects on growth, liver histology, or other health parameters, underscoring their safety. The findings highlight SeOX and CHS as practical, sustainable solutions to counteract AFB_1_ toxicity in poultry farming, aligning with food safety regulations while safeguarding animal health and productivity. This work advances strategies for mycotoxin risk management in agriculture through non-toxic, binder-based interventions.

Sinelnikov et al. (contribution 1) developed a sustainable enzymatic method to degrade AFB_1_ in cereal grains using a recombinant oxidase (AFO) from *Armillaria tabescens*. Engineering *Pichia pastoris* with a chimeric signal peptide enhanced extracellular AFO production (0.3 g/L). AFO degraded 80% of AFB_1_ in model solutions under optimal conditions (pH 6.0, 30 °C) within 72 h. In *A. flavus*-inoculated wheat and corn, AFO reduced AFB_1_ by over twofold in moderately contaminated and ~40% in heavily contaminated samples. The study highlights AFO’s promise as a scalable, eco-friendly bioremediation tool, especially for moderate contamination levels.

Javed et al. (contribution 11) reviewed innovative strategies to combat *Aspergillus* contamination and aflatoxin production in global grain and nut supplies. They highlighted atmospheric cold plasma (ACP), a non-thermal, residue-free technology that effectively inactivates *Aspergillus* spp. and degrades aflatoxins. The review also introduces a recombinant aflatoxin-degrading oxidase with strong detoxification potential in contaminated grains. These physical and biological interventions offer sustainable, scalable solutions for aflatoxin control, addressing food safety challenges and aligning with the demand for minimally processed, safe food.

Glesener et al. (contribution 5) investigated X-ray irradiation as a sterilization method to manage *A. flavus* and its associated AFB_1_ in naturally contaminated maize. Irradiation up to 3.0 kGy effectively eliminated viable spores, as confirmed by culture-based methods. However, AFB_1_ levels remained unchanged, showing that the toxin is not degraded while fungal viability is reduced. qPCR analysis confirmed persistent fungal DNA post-treatment, indicating that the method targets viability, not presence. This safe, reproducible approach is valuable for handling contaminated samples in research settings without altering toxin levels. Though unsuitable as a standalone food decontamination method, it supports integrated strategies for comprehensive mycotoxin control.

## 4. AFB_1_ Toxicity and Health Effects

Chen et al. (contribution 6) presented a comprehensive multi-omics analysis of AFB_1_-induced intestinal cytotoxicity using SW480 human colorectal cells. Exposure to 50 µM AFB_1_ for 72 h caused significant cellular damage via oxidative stress, calcium overload, mitochondrial dysfunction and lipid metabolism disruption. Integrated transcriptomic, proteomic, and metabolomic data revealed activation of oxidative stress pathways and lipid dysregulation. Molecular dynamics simulations identified sterol carrier protein-2 (SCP2) as a key mediator, suggesting its role in amplifying AFB_1_ toxicity through lipid transport. This study provides new insight into the gastrointestinal effects of AFB_1_, expanding the focus beyond hepatic toxicity and highlighting the power of multi-omics in unraveling complex mycotoxin responses.

Yu et al. (contribution 3) examined AFB_1_-induced hepatotoxicity in ducks, focusing on liver cholestasis and dietary mitigation strategies. Over four weeks, exposure to 90 µg/kg AFB1 caused significant oxidative stress. It disrupted bile acid metabolism, indicated by increased liver malondialdehyde (MDA) and total bile acid (TBA) levels and reduced antioxidant enzyme activity. AFB_1_ upregulated cytochrome P450 family 7 subfamily A member 1 (CYP7A1) and cytochrome P450 family 8 subfamily B member 1 (CYP8B1), key enzymes in bile acid synthesis, while downregulating ATP-binding cassette subfamily B member 11 (BSEP), a critical bile acid transporter. Nutritional supplements, especially taurine and emodin, effectively countered these effects by restoring bile acid homeostasis and modulating gene expression. The study deepens the understanding of AFB_1_-induced liver damage and highlights the potential of dietary interventions to protect poultry from contaminated feed.

Choi et al. (contribution 7) presented a comprehensive review of the adverse effects of AFB_1_ on intestinal health and growth performance in monogastric animals, particularly poultry and swine. AFB_1_-contaminated feed impairs gut microbiota balance, induces oxidative stress and immune activation, and compromises intestinal morphology and barrier function, reducing nutrient absorption and growth. A meta-analysis revealed a dose-dependent decline in average daily gain, with pigs showing greater sensitivity than chickens. The review highlights the small intestine as a primary site of AFB_1_ damage. It emphasizes the efficacy of multi-component detoxifying agents, such as adsorbents and bio-transforming microbes like *Bacillus subtilis*, in mitigating these effects. By restoring gut integrity and promoting growth, these integrated dietary strategies offer practical and scalable solutions for reducing AFB_1_’s impact on livestock health and productivity. This approach has broad implications for feed safety and food security.

## 5. Fungal Biology and Aflatoxin Biosynthesis Regulation

Wang et al. (contribution 4) presented compelling evidence for the antifungal and anti-aflatoxigenic efficacy of rhein, a natural anthraquinone compound derived from *Rheum palmatum* L., against *A. flavus*. Rhein treatment at 50 µM inhibited mycelial growth and spore formation while reducing AFB_1_ production by 87.2% in vitro. RNA-seq analysis revealed that rhein interferes with essential cellular processes, including energy metabolism, oxidative stress response, and cell wall integrity, leading to mitochondrial dysfunction, ATP depletion, and elevated ROS levels. These disruptions compromise fungal viability and toxin biosynthesis. Testing against peanut and maize seeds demonstrated strong in situ activity, with up to 90.4% reduction in spore growth and 99.43% reduction in AFB_1_ levels. This dual-action mechanism underscores rhein’s potential as a sustainable, natural strategy for controlling aflatoxigenic fungi and enhancing crop safety in post-harvest and storage systems.

Zhang et al. (contribution 2) explored the functional significance of the chitin deacetylase (CDA) gene family in *A. flavus*, revealing novel insights into fungal development and aflatoxin regulation. Although the six annotated CDA homologs (Cda1–Cda6) showed limited influence on chitin content or cell wall integrity, the targeted deletion of cda6 *(∆cda6*) produced a distinct phenotype, marked by enhanced conidiation, reduced mycelial growth, and significantly elevated aflatoxin production. Additionally, *∆cda6* colonization of host seeds was impaired. Subcellular localization studies confirmed that Cda6 functions within the cell wall and requires conserved CBD1 and CBD2 domains for its regulatory activity. Interestingly, the hyper-aflatoxigenic nature of *∆cda6* suggests that cda6 exerts a previously unrecognized role in modulating toxin biosynthesis, independent of classical chitin modification. These findings redefine the functional landscape of CDA proteins in fungal pathogens and highlight cda6 as a promising molecular target for mitigating aflatoxin contamination through innovative biocontrol strategies that avoid direct disruption of structural cell wall components.

## 6. Host Susceptibility and Crop/Public Health Risks

Elamin et al. (contribution 10) examined the susceptibility of *Ziziphus jujuba* var. *spinosa* (jujube) fruits and seeds to aflatoxin contamination by *A. flavus*, emphasizing the influence of fruit maturation and seed morphology. Mid-mature fruits were most prone to aflatoxin accumulation. Following fungal inoculation, internal seed tissues showed significant toxin levels even when physical barriers such as the exocarp, mesocarp, and endocarp remained intact. A key finding was the role of hilar region morphology in mediating fungal penetration and subsequent aflatoxin accumulation, accounting for contamination variability within seeds of the same developmental stage. The study reveals that *A. flavus* can breach jujube’s structural defenses and colonize internal tissues, positioning hilar morphology as a critical susceptibility factor. These insights underscore the importance of integrating seed structural analysis and maturation-stage monitoring into food safety strategies for jujube-based medicinal products, aiding in preventing aflatoxin contamination in this high-value crop.

Chelenga et al. (contribution 13) presented a comprehensive review assessing the human health risks associated with aflatoxin residues in chicken products from birds consuming contaminated feed. Based on 33 global studies (1984–2023), this review shows that less than 1% of aflatoxins present in poultry feed are transferred to edible tissues. The average concentrations detected were 2.0 µg/kg in tissues and 0.3 µg/kg in eggs. Concentrations exceeding 20 µg/kg were detected in only 0.6% of cases and were limited to instances where feed contamination levels were above 300 µg/kg, the threshold set by the FDA for poultry feed. The findings support the concept of chickens acting as “biological filters”, efficiently metabolizing or sequestering aflatoxins and limiting human dietary exposure. Despite prolonged feeding (>100 days), residue increases were modest and manageable. The study emphasizes that current feed regulations are primarily designed to protect poultry health rather than human consumers, for whom the risk remains minimal. The authors advocate for re-evaluating aflatoxin feed thresholds and propose exploring the filtering capacities of other poultry species, such as ducks and geese, to utilize contaminated grains safely. This review provides reassurance that chicken products pose a negligible aflatoxin exposure risk, even in regions burdened by feed contamination.
